# Structural and Magnetic Studies of Bulk Nanocomposite Magnets Derived from Rapidly Solidified Pr-(Fe,Co)-(Zr,Nb)-B Alloy

**DOI:** 10.3390/ma13071515

**Published:** 2020-03-26

**Authors:** Katarzyna Pawlik, Piotr Pawlik, Jerzy J. Wysłocki, Waldemar Kaszuwara

**Affiliations:** 1 Department of Physics, Faculty of Production Engineering and Materials Technology, Częstochowa University of Technology, Al. Armii Krajowej 19, 42-200 Częstochowa, Poland; piotr.pawlik@pcz.pl (P.P.); jerzy.wyslocki@pcz.pl (J.J.W.); 2 Faculty of Materials Science and Engineering, Warsaw University of Technology, ul. Wołoska 141, 02-507 Warsaw, Poland; Waldemar.Kaszuwara@pw.edu.pl

**Keywords:** bulk metallic glasses, hard magnetic materials, RE-Fe-B magnets, rapid solidification, devitrification annealing, Rietveld analysis, magnetic properties, miniature magnets, bulk nanostructured magnets

## Abstract

In the present study, the phase constitution, microstructure and magnetic properties of the nanocrystalline magnets, derived from fully amorphous or partially crystalline samples by annealing, were analyzed and compared. The melt-spun ribbons (with a thickness of ~30 µm) and suction-cast 0.5 mm and 1 mm thick plates of the Pr_9_Fe_50_Co_13_Zr_1_Nb_4_B_23_ alloy were soft magnetic in the as-cast state. In order to modify their magnetic properties, the annealing process was carried out at various temperatures from 923K to 1033K for 5 min. The Rietveld refinement of X-ray diffraction patterns combined with the partial or no known crystal structures (PONKCS) method allowed one to quantify the component phases and calculate their crystalline grain sizes. It was shown that the volume fraction of constituent phases and their crystallite sizes for the samples annealed at a particular temperature, dependent on the rapid solidification conditions, and thus a presence or absence of the crystallization nuclei in the as-cast state. Additionally, a thermomagnetic analysis was used as a complementary method to confirm the phase constitution. The hysteresis loops have shown that most of the samples exhibit a remanence enhancement typical for the soft/hard magnetic nanocomposite. Moreover, for the plates annealed at the lowest temperatures, the highest coercivities up to ~1150 kA/m were measured.

## 1. Introduction

Hard magnetic materials have proven to be indispensable in modern technology. They can be characterized by few macroscopic parameters, including: (i) the coercivity field (*_J_H_c_*)—which refers to the maximum reversed magnetic field up to which the hard magnetic specimen resist demagnetization; (ii) the polarization remanence (*J_r_*)—the value of magnetic polarization measured at zero external magnetic field for the initially magnetically saturated specimen; (iii) maximum magnetic energy product (*BH*)_max_—the amount of magnetic energy that can be stored in the magnet and (iv) the Curie temperature (*T_C_*)—the temperature of the transition from ferro- to paramagnetic state of the ferromagnetic material.

Over the past half century, the RE-Fe-B-type (RE—rare earth element) hard magnetic alloys have become commonly used in different areas of life, including in automotive, electronic, and household appliances [1]. In particular, the green energy related applications in hybrid vehicles or wind turbines triggered a burst of interest in both the processing routes, as well as in alteration of the alloy composition [2,3]. The high efficiency of miniature magnetic components of various devices is caused by the presence of the RE_2_Fe_14_B phase, that reveals high magnetic anisotropy, leading to high coercivity (*_J_H_c_*) and maximum magnetic energy product (*BH*)_max_. The comprehensive studies of basic physical properties of the RE_2_Fe_14_B compounds were presented in [4,5]. It was shown that the RE_2_Fe_14_B phase can be formed for many RE elements, which differs in the saturation polarization (*J_s_*), magnetic anisotropy field (*H_A_*) and Curie temperature (*T_C_*). The most important magnets in this group are the sintered magnets [6] and melt-spun ribbon [7], made of the Nd-Fe-B alloy. For this type of magnets, *_J_H_c_* can reach up 1100 kA/m, *J_r_*—of ~0.8 T and (*BH*)_max_ of ~122kJ/m^3^. The hard magnetic Pr_2_Fe_14_B phase has similar magnetic properties to those of the Nd_2_Fe_14_B [8,9]. Furthermore, the Pr_2_Fe_14_B phase reveals higher magnetocrystalline anisotropy [10] and does not undergo a spin reorientation down to 25 K [11]. This allows one to sustain good magnetic properties at low temperatures.

A considerable effort was put into the development of RE-Fe-B alloys by appropriate micro-alloying. It can play a beneficial role in the formation of the appropriate phase composition and microstructure of the RE-Fe-B-type alloys. Particularly, the admixture of some refractory elements (e.g., Zr, Nb or W) can be used for tailoring their glass forming abilities (GFA) [12,13,14,15]. The micro-alloying of RE-Fe-B alloys with the use of Ti and C can modify the demagnetization curves, by suppressing the growth of crystallites during annealing [12]. This also results in *J_r_*, *_J_H_c_* and (*BH*)_max_ enhancement.

Several methods are used for fabrication of the RE–Fe–B magnets i.e., sintering [6], mechanical alloying [16], HDDR (hydrogenation, decomposition, desorption and recombination) [17] or rapid solidification [18,19]. In particular, the rapid solidification techniques offer many possibilities in processing magnetic materials. These allow one to fabricate the amorphous structure of the alloy, which can be devitrified in a controllable way to obtain the appropriate microstructure and magnetic properties of the final product.

A large technological challenge is the production of full density bulk nanostructured magnets. In order to produce such magnetic materials, two different approaches can be adopted. First one is the consolidation of nanostructured powders made by mechanical alloying or by crushing the melt-spun ribbons. In conventional consolidation processes (such as sintering or hot pressing), the growth of grain leads to significant deterioration of their magnetic properties [19]. Furthermore, these magnets are isotropic, due to difficulties in the arrangement of grains during compaction. Therefore, in order to reduce those disadvantages, some novel consolidation techniques are used, including, for example: the spark plasma sintering [20,21,22,23] and the shock wave compaction [24].

The second, and definitely novel approach, is the devitrification annealing of bulk glassy precursors, that can by produced by injection-casting or suction-casting techniques [25,26] in various forms, for example rods, tubes or plates. The advantages of this approach are: (i) much higher corrosion resistance, due to the formation of a fully dense structure, (ii) a lack of dilution of magnetic phase within the binding polymer (which results in the reduction of *J_r_* and (*BH*)_max_) and (iii) reduction of the processing time, thus a reduction of the final costs of the product. However, the design of the composition that secures a good GFA for casting bulk specimens is always a challenge. A good example of such task is the (Pr,Dy)_4.5_(Fe, Co)_73.5_Zr_1_B_20_ alloy, for which it is possible to cast up to 1 mm diameter rods and up to 3 mm outer diameter tubes [27]. A disadvantage of this composition manifests itself in the low content of the Pr_2_Fe_14_B (2:14:1) phase in the alloy subjected to heat treatment. Further studies allowed the fabrication of the Pr_9_Fe_60_Co_13_B_14_Ti_3_Zr_1_ alloy containing 9 at% of Pr [28], that can be directly cast in the form of nanocrystalline 1mm dia. rods. A presence of larger content of Pr resulted in significant increase of *_J_H_c_*. Our further investigations of Pr-Fe-B-based alloys led us to study the GFA for the melt-spun ribbons [29] and suction-cast rods and plates of Pr_9_Fe_50+x_Co_13_Zr_1_Nb_4_B_23−x_ (x = 0, 2, 5, 8) alloys [30]. In this group, the Pr_9_Fe_50_Co_13_Zr_1_Nb_4_B_23_ alloy seems to be the most promising, due to its good glass forming abilities, allowing one to suction-cast up to 1 mm thick amorphous plates. Therefore, our current studies are focused on the investigations of the phase constitution, microstructure and magnetic properties of the melt-spun ribbons, and suction-cast plates of this alloy, subjected to annealing at various temperatures.

## 2. Materials and Methods

The ingot samples of the Pr_9_Fe_50_Co_13_Zr_1_Nb_4_B_23_ alloy were produced by arc-melting of the high purity constituent elements, with the addition of the Fe-B pre-alloy. Subsequently, the melt-spun ribbon (~30 µm thick) and suction-cast 0.5 mm and 1 mm thick plates were produced. In order to modify their magnetic properties, the annealing process was carried out at various temperatures (*T_a_*), from 923 K to 1033 K for 5 min. For this purpose, the samples were sealed off in the quartz tubes under the low pressure of Ar and subjected to annealing in the lab resistance furnace. The *T_a_* were chosen, based on differential scanning calorimetry measurements carried out on fully amorphous ribbons [29].

The X-ray diffraction (XRD) studies were performed in order to determine the phase constitution of the investigated specimens. Furthermore, the Rietveld refinement was used in order to quantify the amounts of constituent phases in samples annealed at various temperatures and having different shapes. Additionally, the application of the partial or no known crystal structures (PONKCS) method allowed one to estimate the amount of remaining amorphous phase within the samples subjected to annealing. The crystallite sizes, as well as the unit cell parameters, were determined, based on the Rietveld refinement. The XRD patterns were collected using the Bruker D8 Advance diffractometer (Bruker AXS GmbH, Karlsruhe, Germany), with CuK_α_ radiation equipped with a LynxEye detector (linear focus of 25 mm, primary beam divergence slit—0.6 mm), with Soller slits on a primary and diffracted beam. The measurements were performed in the Bragg–Brentano configuration, with the Ni K_β_ filter on the detector side. The 2θ step size was 0.02 deg and step time 5 s. The Rietveld refinements were performed using DIFFRAC SUITE TOPAS 4.2 software (Bruker AXS GmbH, Karlsruhe, Germany).

The temperature dependences of magnetization were measured using the Faraday balance, in the temperature range from 300 K to 820 K, at an external magnetic field of 0.1 T. It was used as a complementary method, to confirm the phase constitution. Measurements of magnetic hysteresis loops allowed one to determine the optimal annealing condition for obtaining the highest magnetic parameters. The measurements were performed using a LakeShore 7307 VSM magnetometer operating at room temperature, in external magnetic fields up to 1590 kA/m.

## 3. Resultsand Discussion

**Figure 1:**
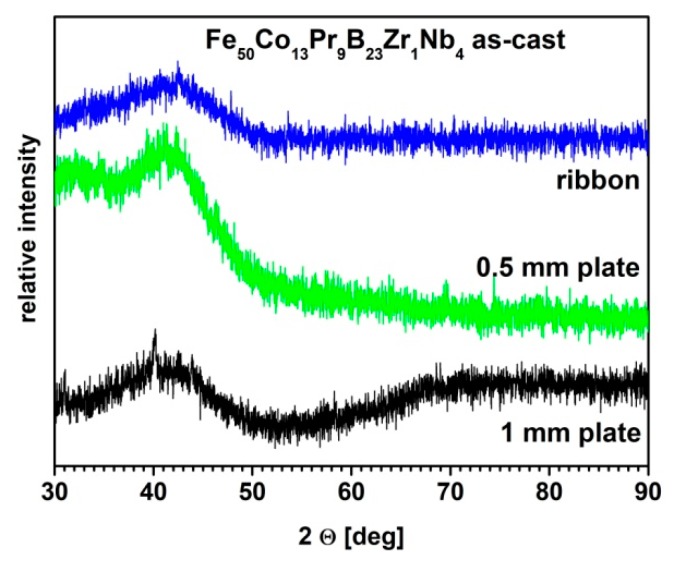
The X-ray diffraction (XRD) patterns measured for the melt-spun ribbon and the suction-cast 0.5 mm and 1 mm thick plates.

In Figure 1, the XRD patterns measured for the melt-spun ribbon, as well as for the suction-cast 0.5 mm and 1 mm thick plates are presented.

These studies have shown that melt-spun ribbons were fully amorphous. It was also confirmed by the measurement of the hysteresis loop, which was typical for the soft magnetic materials. In the case of the 0.5 mm thick plate, the XRD pattern revealed a characteristic bump for the amorphous phase, however the presence of small intensities’ diffraction peaks was possible, but not clear, due to the relatively high background. A lack of pronounced diffraction peaks might be related to a weight fraction of the precipitating crystalline phase which is too low to be detected by the XRD method. In the case of the 1 mm thick plate, except for the amorphous bump, very low intensity diffraction peaks were detected. Nevertheless, these studies proved the relatively good GFA of the Fe_50_Co_13_Pr_9_Zr_1_Nb_4_B_23_ alloy. In order to modify the microstructure and magnetic properties, a further annealing process was performed in temperatures (*T_a_*), ranging from 923 K to 1033 K, for 5 min. The annealing conditions were adjusted based on the differential scanning calorimetry (DSC) measurements [29], which have shown a crystallization process manifested by a wide crystallization peak, for which the beginning of crystallization occurs at *T_x_* of 931 K and the maximum of crystallization—at *T_p_* = 944 K.

**Figure 2:**
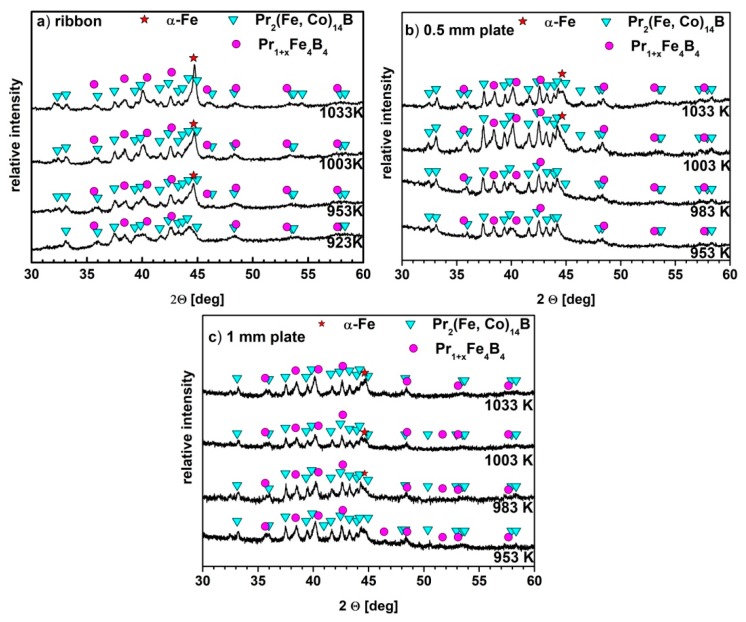
The XRD patterns measured for (**a**) the ribbons, (**b**) 0.5 mm and (**c**) 1 mm plates, annealed at various temperatures for 5 min.

The XRD studies of the annealed samples are presented in Figure 2. It was shown that the annealing of a thin ribbon at 923 K (Figure 2a) resulted in significant crystallization of the sample. Although the *T_x_* measured for the ribbon is higher than 923 K, one has to consider that the DSC measurements were performed dynamically, with a heating rate of 10 K/min. The shift of the crystallization temperature to higher values with the increase of the heating rate is characteristic for the DSC measurements [31]. Thus, in the case of static annealing conditions, the crystallization can begin at lower temperatures. The qualitative XRD analyses revealed a presence of the hard magnetic Pr_2_(Fe,Co)_14_B (2:14:1) and paramagnetic Pr_1+x_Fe_4_B_4_ (1:4:4) crystalline phases in all ribbon specimens subjected to annealing. Furthermore, the increase of annealing temperature caused an increase of the diffraction peak intensities, corresponding to the soft magnetic α-Fe phase. In all annealed 0.5 mm and 1 mm thick plates, the same crystalline phases as those detected for the ribbon specimens were revealed.

**Figure 3:**
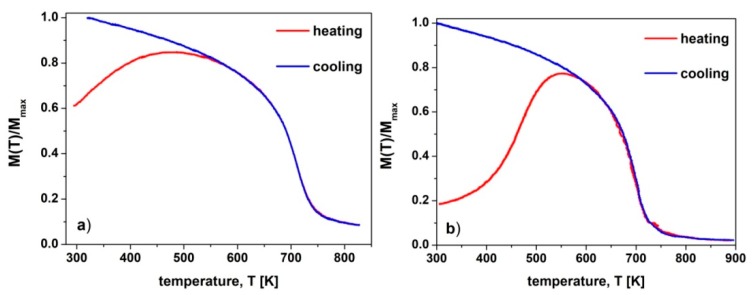
The temperature dependences of reduced magnetization *M*(*T*)/*M*_max_, measured under the heating and cooling of (**a**) the ribbons and (**b**) 0.5 mm plates annealed at 953 K for 5 min.

However, in case of multiphase nanocrystalline materials, the unambiguous phase identification is a difficult task, for several reasons. The most important are a strong overlapping, the angular widening due to the nanocrystalline structure, and the low intensity of peaks corresponding to the constituent phases. These let us to consider a further quantitative analysis of the XRDs using the Rietveld refinement method. The crucial precondition for obtaining a successful fit is a reasonable initial model of the material’s phase structure. This allows one to avoid the false local minima of fit. To provide such a condition, the complementary studies of the temperature dependences of magnetization *M*(*T*) were performed for as-cast and annealed specimens. The measurements were carried out, both under the heating and cooling conditions, at low external magnetic field (*µ*_0_*H* =0.1 T), therefore the measured value of magnetization is not the saturation magnetization. The heating and cooling *M*(*T*) curves have different shapes (Figure 3). In the case of *M*(*T*) curves measured on the heating of the annealed samples, the increase of temperature causes a decrease of the anisotropy constant in the hard magnetic Pr_2_Fe_14_B phase [32,33], and thus the alignment of the magnetic domains along the external magnetic field. As a result, the significant rise of magnetization with the temperature is observed below the Curie point (*T_C_*) of the Pr_2_Fe_14_B phase. It could cause difficulties in distinguishing the characteristic steps at *T_C_* coming from the magnetic phase transitions of other ferromagnetic phases.

**Figure 4:**
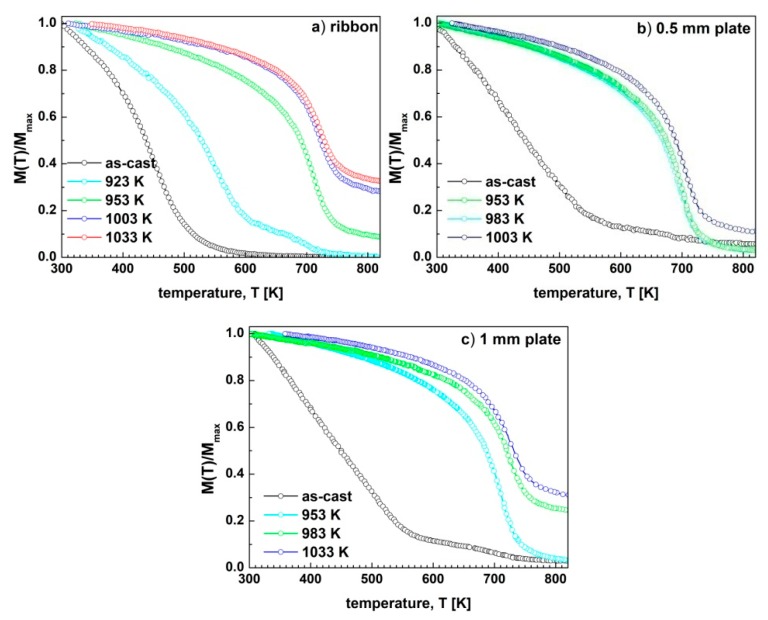
The temperature dependences of reduced magnetization *M*(*T*)/*M_max_* for (**a**) the ribbons, (**b**) 0.5 mm and (**c**) 1 mm plates, annealed at various temperatures for 5 min.

Therefore, the *M*(*T*) curves measured under heating for the as-cast specimens and under cooling conditions for the annealed samples are presented in Figure 4. The *T_C_* was determined as the minimum of the first derivative of magnetization, with respect to the temperature, and its values are collected in Table 1. However, in the case of the amorphous phase, the magnetization gradually decreases with the increase of the temperature. Therefore, in order to determine the *T_C_*, the *M*^1/*β*^(*T*) curves were constructed, where *β* = 0.36 is an effective critical exponent [34]. In this procedure, *T_C_* were determined by the extrapolation of the linear part of *M*^1/*β*^(*T*) curve to *M* =0.

For the as-cast ribbon (Figure 4a), the heating and cooling curves look similar and the magnetization gradually decreases with the increase of the temperature, and at ~515 K the sample becomes paramagnetic. The annealing of the ribbon at 923 K resulted in a significant increase of the *T_C_* corresponding to the amorphous component phase, which is still present in the sample. This increase is related to the change of the chemical composition of the amorphous phase, due to a formation of the hard magnetic and paramagnetic crystalline components. The presence of the hard magnetic phase was evidenced by the second step in the *M*(*T*) curve. This result is in agreement with the XRD studies, which have proven the presence of hard magnetic Pr_2_(Fe,Co)_14_B, and paramagnetic Pr_1+x_Fe_4_B_4_ phases. Annealing of the ribbon at 953K and higher temperatures resulted in a significant change of the *M*(*T*) curves. A ferro- to paramagnetic transition occurs at ~708 K. Such high *T_C_* measured for the hard magnetic 2:14:1 phase can be attributed to the partial occupation of the Fe positions in the unit cell by the Co atoms. Such an effect was also reported in [29,35] for Pr-Fe-Co-B alloys. An increase of *T_C_* with the rise of the annealing temperature might be attributed to the Co substitution changes in 2:14:1 phase. Additionally, the thermomagnetic measurements have confirmed the presence of α-Fe phase, for the specimens annealed at 953 K, 1003 K and 1033 K. Due to the limited range of the measurement temperatures, the α-Fe ferro- to paramagnetic transition is not visible on these curves, but the presence of this phase is indicated by the high magnetic moment of the samples at temperatures higher than 750 K. The *M*(*T*) curves also confirmed the increase of the volume fraction of the α-Fe phase, with the rise of the *T_a_*.

Partial crystallization of both the as-cast 0.5 mm and 1 mm thick plates had its reflection in the two-step *M*(*T*) curves measured for these samples (Figure 4b,c). This effect was more pronounced for the 1 mm thick plates, where the cooling rate during rapid solidification of the samples was much lower than this for the 0.5 mm thick plates. On the other hand, the annealing of the 0.5 mm thick plate at 953 K and 983 K resulted in the formation of the only one ferromagnetic 2:14:1 phase, without precipitation of α-Fe. An increase of the *T_a_* up to 1003 K resulted in the additional formation of the α-Fe phase, that is reflected in the significant value of the magnetization at temperatures higher than 750 K. In the case of the 1 mm thick plates, the α-Fe phase is present for the sample annealed at 983 K, and its fraction increases with the increase of *T_a_*.

**Figure 5:**
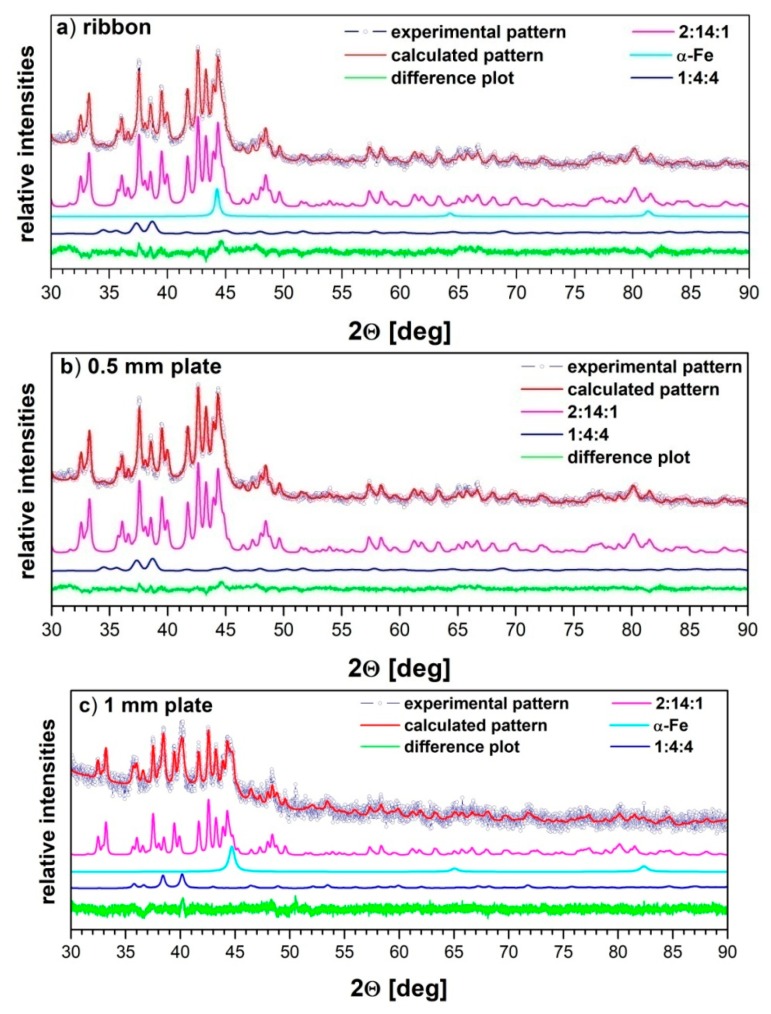
The Rietveld refinement output plots with marked experimental and calculated diffraction patterns, difference curves and constituent phases for Pr_9_Fe_50_Co_13_Zr_1_Nb_4_B_23_ alloy (**a**) ribbons, (**b**) 0.5 mm plates and (**c**) 1 mm plates, annealed at 953 K for 5 min.

Examples of Rietveld refinements carried out for the annealed ribbon and plates of various thicknesses were shown in Figure 5. The criteria of fit [36] are collected in Table 2. In the starting structural model used in the Rietveld refinement, the presence of two crystalline phases 1:4:4 and 2:14:1 for all annealing temperatures were considered. The α-Fe and amorphous phases were also incorporated into the model, when positive indication of their presence was demonstrated by the thermomagnetic studies. Quantification of the amorphous phase was possible with the use of the PONKCS method [37]. In this approach, the amorphous component is modeled as a group of peaks (so called “peak phase”), with its specific ZMV parameter. In the case of known crystalline phases, this parameter is related to the mass (ZM) and the volume (V) of its unit cell. However, for the amorphous phase, such structural details cannot be specified. Therefore, the value of the ZMV parameter for such a phase is derived from the diffraction pattern measured for the mixture of the known amounts of amorphous phase and well-characterized internal standard. This ZMV value has no physical meaning, but serves as a calibration value for the calculations of the amorphous phase concentration.

The Rietveld refinement was used to calculate the weight fractions (*W_f_*) and the unit cell parameters of constituent phases, and the volume weighted coherently diffracting domain sizes (*L_vol_*) that are the measure of the crystallite sizes (*d*). In Table 3, the starting values of the lattice parameters for the crystalline phases included in the model, together with their refined values, were presented. As one can see from Table 3, the addition of Co to the alloy composition resulted in the reduction of the unit cell parameters of the 2:14:1phase. It has been frequently reported that the addition of Co to the hard magnetic RE-Fe-B alloys results in changes of their magnetic properties, without the change of the 2:14:1 phase structure type [38,39,40,41]. The Co atoms take the Fe sites in the unit cell of the 2:14:1 phase, resulting in a monotonic decrease of the unit cell parameters. This leads to the change of the interatomic distances, thus affecting the exchange interactions. It was proven by neutron diffraction analysis [32] and Mössbauer spectroscopy [42], that Co atoms preferentially take a *4e* position in the unit cell of the 2:14:1 phase, while other positions (*16k*_1_, *16k*_2_, *8j*_1_ and *4c*) are randomly occupied by Co. It has a crucial influence on *T_C_* [29,35,38]. It is worth mentioning that the unit cell parameter *c* calculated for 1:4:4 phase also decreased, while the α-Fe unit cell remains almost unchanged.

**Figure 6:**
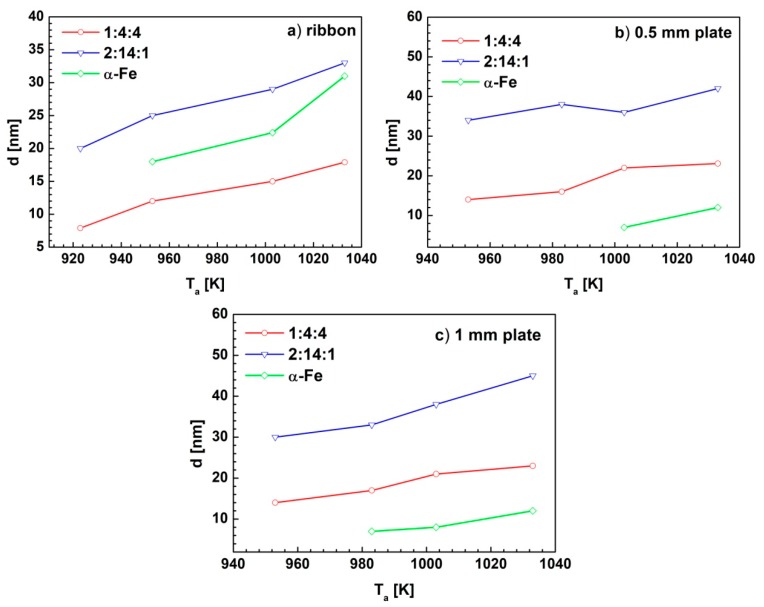
The dependences of the crystallite size (*d*) of constituent phases on annealing temperature for the Pr_9_Fe_50_Co_13_Zr_1_Nb_4_B_23_ alloy (**a**) ribbons, (**b**) 0.5 mm plate and (**c**) 1mm plate.

**Figure 7:**
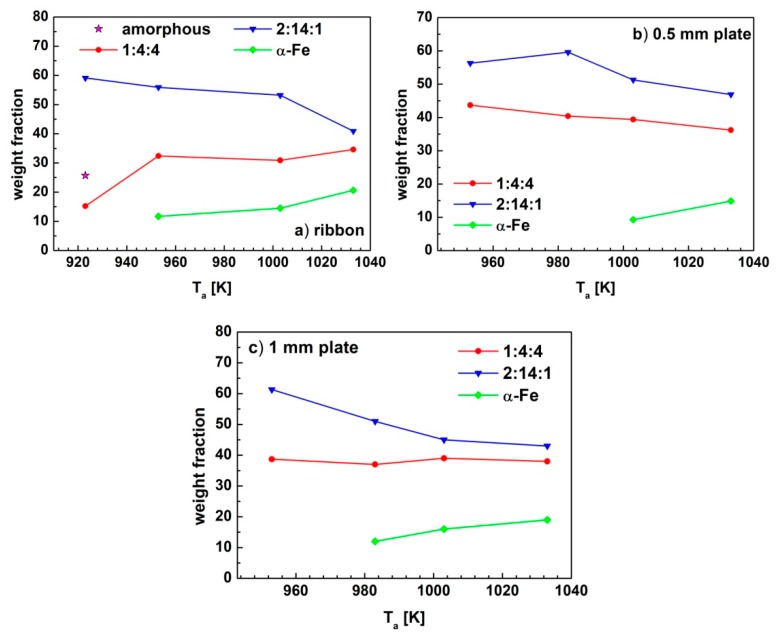
The dependences of the weight fractions (*Wf*) of constituent phases on annealing temperature for the Pr_9_Fe_50_Co_13_Zr_1_Nb_4_B_23_ alloy (**a**) ribbons, (**b**) 0.5 mm plate and (**c**) 1 mm plate.

The dependences of calculated average diameters of nanocrystallites (*d*) and the weight fractions (*Wf*) of the component phases on the annealing temperature were shown in Figure 6 and Figure 7, respectively. The refined values of these parameters were collected in Table 4.

For all samples, the rise of the *T_a_* led to an increase in the average grain diameters of the constituent crystalline phases (Figure 6). The Rietveld refinement has shown that the hard magnetic phase forms the largest crystallites during annealing, with the diameters changing from 20 to 40 nm. Comparable sizes of crystallites (from 18 to 30 nm) were calculated for the α-Fe phase in the ribbon specimens. In case of bulk samples, the crystallites of α-Fe phase were much smaller, with average diameters lower than 10 nm. The weight fractions of constituent phases change in various ways with the increase of the annealing temperature (Figure 7). In the case of thin ribbon, the largest fraction of the hard magnetic 2:14:1 phase is formed for the sample annealed at 923 K. For this sample, the amorphous, as well as paramagnetic 1:4:4 phases, were observed. An increase in the annealing temperature led to a gradual decrease in the *W_f_* of the hard magnetic phase, in favor of the paramagnetic 1:4:4 and/or the soft magnetic α-Fe phases (which appear at the annealing temperatures above 953 K). For the ribbons annealed at temperatures higher than 923 K, no amorphous phase was found.

**Figure 8:**
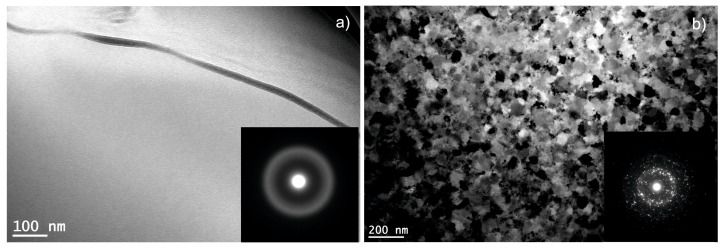
The transmission electron micrographs (TEM), together with the electron diffractograms (inset), obtained for the 0.5 mm thick plate in (**a**) as-cast state and (**b**) subjected to annealing at 983 K for 5 min.

The examples of transmission electron micrographs (TEM), together with the electron diffraction patterns obtained for the 0.5 mm thick plates in as-cast state and the one subjected to annealing at 983 K, are presented in Figure 8. Due to the characteristics of the TEM studies (the observations were carried out only on the limited areas of the sample), the crystallites nucleated during rapid solidification of the bulk sample were not revealed. Furthermore, its electron diffraction image is typical for the amorphous phase. In case of the annealed specimen, the microstructure is heterogeneous, with crystal particles of different sizes. Additionally, the electron diffraction image is typical for the nanocrystalline specimens. The presence of crystal nuclei within the amorphous matrix of the as-cast plate resulted in a growth of crystallites, nucleated during the rapid solidification and simultaneous nucleation of new ones during the heat treatment. The average crystallite sizes are similar to those calculated using whole powder pattern fitting in the Rietveld refinement procedure.

**Figure 9:**
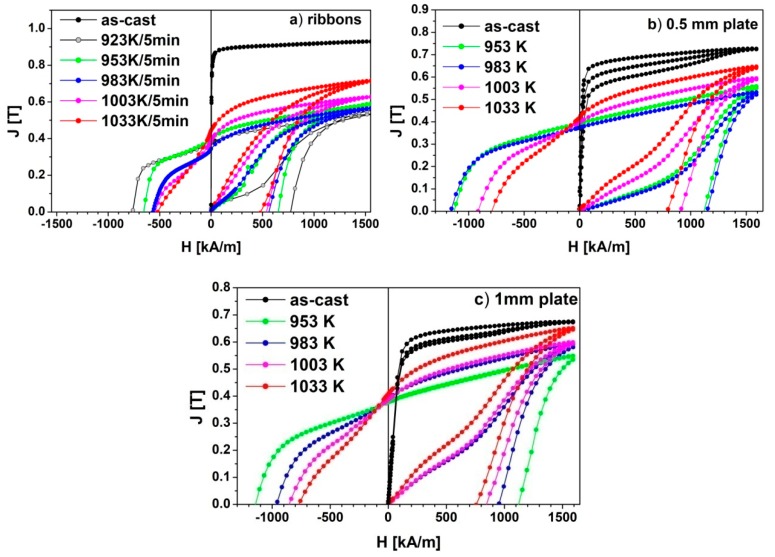
The hysteresis loops measured for (**a**) ribbons, (**b**) 0.5 mm plate and (**c**) 1 mm plate of Pr_9_Fe_50_Co_13_Zr_1_Nb_4_B_23_ alloy, annealed at various temperatures.

The changes in the phase constitution with the annealing temperature are reflected in the shapes of the magnetic hysteresis loops (Figure 9). For the as-cast ribbon, the shape of the hysteresis loop confirms its fully amorphous structure (Figure 9a). Annealing of the ribbon at 923 K allowed one to reach the coercivity value *_J_H_c_* of 762 kA/m, which is the highest for the annealed ribbons. Heat treatment of ribbons at higher temperatures resulted in the gradual decrease of *_J_H_C_* and the increase of the saturation polarization *J_s_*. This can be related mainly to the increase of the weight fraction of the α-Fe phase, with the increase of the annealing temperature and the growth of crystallites of the 2:14:1 phase, that was shown by the Rietveld refinement. The highest maximum energy product (*BH*)_max_ and remanence *J_r_* were measured for the ribbon annealed at 953 K.

**Figure 10:**
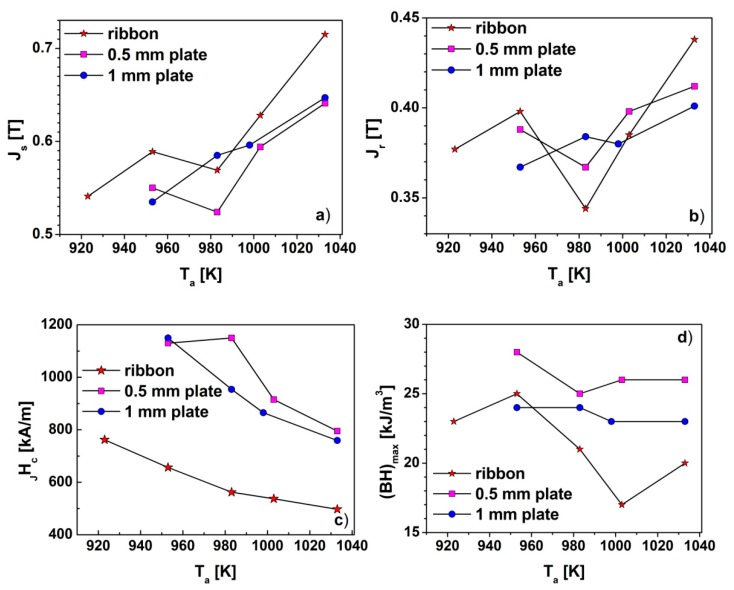
The dependences of magnetic parameters’ values: (**a**) saturation polarization *J_s_*, (**b**) polarization remanence *J_r_*, (**c**) coercivity field *_J_H_c_* and (**d**) maximum magnetic energy product (*BH*)_max_ on *T_a_*.

More complex shapes of the hysteresis loops, measured for both the as-cast 0.5 mm and 1 mm thick plates (Figure 9b,c), correspond to their partially crystalline structure. Here the wasp-wasted shapes of hysteresis loops are typical for the specimens, where small fractions of the hard magnetic phase are diluted within the soft magnetic amorphous matrix. In the case of bulk samples, the highest values of the coercivity *_J_H_c_* reaching 1150 kA/m and maximum magnetic energy product (*BH*)_max_=25 kJ/m^3^ were measured for the 0.5 mm and 1 mm thick plates annealed at 983K and 953K, respectively. These samples also had the lowest saturation polarization. The increase in the saturation polarization of samples annealed at higher temperatures can be attributed to the increase in the content of the α-Fe phase. The magnetic parameters determined from the hysteresis loops, i.e., magnetic polarization remanence *J_r_*, saturation polarization *J_s_*, coercivity field *_J_H_c_* and maximum magnetic energy product (*BH*)_max_, were collected in Table 5, and their dependences on *T_a_* are presented in Figure 10.

High values of *J_r_*/*J_s_* > 0.6 for all annealed specimens suggest the presence of the spring effect in all investigated magnets. The highest values of *J_s_* = 0.715 T were measured for the ribbon annealed at 1033 K. However, the *J_s_*(*T_a_*) dependences are similar for all specimens. The rise of *J_s_* with *T_a_* can be attributed to the increase of the volume fraction of the α-Fe phase. In addition, for all investigated ribbons, *_J_H_c_* were lower than that measured for the bulk specimens. It also has an impact on the (*BH*)_max_ values, which were smaller for annealed ribbon. The highest *_J_H_c_* and (*BH*)_max_ were measured for the 0.5mm thick plates in the whole range of annealing temperatures, despite the fact that *_J_H_c_* was decreasing with the increase of *T_a_*.

## 4. Conclusions

For the Pr_9_Fe_50_Co_13_Zr_1_Nb_4_B_23_ alloy, the 30 µm thick melt-spun ribbon, as well as suction-cast 0.5 mm and 1 mm plates, were produced. XRD studies carried out for these samples have proven the good glass forming ability of this alloy. The ribbon samples were fully amorphous, while in the case of plates, the amorphous phase constitutes the biggest part of the as-cast specimens. The XRD analysis, as well as the magnetic and thermomagnetic measurements, have revealed partial crystallization of both types of as-cast plates. One should notice that the ribbon and plate samples are rapidly solidified at different cooling rates [43]. This has an impact on the initial phase constitution of the samples.

Further heat treatment led to the precipitation of various crystalline phases. The phase constitution however, changed both with the annealing temperature and the thickness of the specimen. For fully amorphous ribbon, it should be assumed that the formation of the crystalline phases occurs simultaneously at a given temperature and their content changes with *T_a_*. For the bulk samples, one can assume that the initial fraction of the crystalline component phases has the main impact on the magnetic parameters of annealed samples. Thus, the heat treatment causes, in the first instance, a growth of crystallites nucleated during the rapid solidification and simultaneous nucleation of new ones. The differences in the phase constitution between ribbon and plate specimens of various thicknesses were revealed, using the Rietveld refinement. Its outcome is consistent with the results of *M*(*T*) measurements. The changes of *T_C_* observed for the 2:14:1 phase in specimens, subjected to annealing at various temperatures, are related to the replacement of Fe atoms by Co in the unit cell of the 2:14:1 phase. This is also reflected in the unit cell parameters calculated for the 2:14:1 phase, based on the XRD studies. The dependences of *_J_H_c_*, *J_r_* and (*BH*)_max_ on the annealing temperature for ribbon and plate samples are closely related to the evolution of the phase constitution with the annealing temperature.

For all annealed specimens except the hard magnetic 2:14:1 phase, the paramagnetic 1:4:4 was also present. Moreover, fractions of ferromagnetic phases (hard magnetic 2:14:1 and soft magnetic α-Fe) are similar for all samples, and likewise change with the annealing temperature (amount of 2:14:1 phase slightly decreases, while α-Fe increases with the rise of *T_a_*). Furthermore, in the case of ribbons, the average diameters of crystallites (*d*) of the 2:14:1 phase increase from 20 nm to 33 nm, with the rise of *T_a_*, while for bulk specimens, the *d* parameter changes from ~30 nm to 45 nm. On the other hand, in ribbons, the *d* values for the α-Fe phase are much higher than those for the bulk specimens. Therefore, lower coercivities measured for the ribbon than those for bulk samples might be explained by lower crystallite sizes of the hard magnetic phase. Similar behavior was observed for the Nd-Fe-B magnets and presented in [5,44]. The highest *_J_H_c_* of 1150 kA/m was measured for the 0.5 mm and 1 mm thick plates, annealed at 983K and 953K, respectively.

The magnetic parameters of nanostructured magnets presented in this work can be compared to other rare-earth (RE) magnets with reduced RE content. An interesting example in this group is the Pr_7_Fe_88_B_5_ alloy with high Fe and low B contents in the form of ribbon [45]. Annealing of this ribbon resulted in the quite high value of *J_r_*, reaching 1.2 T due to the presence of the α-Fe phase, and the very moderate coercivity *_J_H_c_* = 461 kA/m. Similar magnetic properties were measured for the as-quenched melt-spun ribbons of the Pr_9_Fe_79_B_12_ alloy, for which 100 nm grains of Pr_2_Fe_14_B and Fe_3_B phases were found. Their magnetic parameters reached *J_r_* =0.7 T, *_J_H_c_* = 563 kA/m and (*BH*)_max_ = 52 kJ/m^3^ [46].

It should be noted that there are only a few publications of other authors concerning bulk nanostructured RE-Fe-B magnets derived from bulk glassy precursors. The interesting results were obtained for the as-cast 1.5 mm diameter rods of the (Fe_86-x_Nb_x_B_14_)_0.88_Tb_0.12_ alloy, for which *_J_H_c_* reaches up to 5600 kA/m. However, due to the low volume fraction of the Tb_2_Fe_14_B hard magnetic phase, its (*BH*)_max_ reaches only ~13 kJ/m^3^ [47].

The investigated specimens in the form of plates seems to be particularly interesting, due to their potential application. Processing of the specimens in two steps: (i) suction-casting to the partly amorphous structure and (ii) subsequent annealing provides the possibility of obtaining fully dense bulk nanostructured magnets. The advantage of investigated alloy and the usage of a short time manufacturing technique is reflected in the reduction of processing costs. Furthermore, suction-casting allows one to obtain miniature magnets of demanded shapes, that might be used in some small size electromagnetic devices. The only drawback is the need for the use of high purity constituent elements, in order to maintain the good glass forming ability of the alloy. The nanocrystalline magnets reported in the present work were isotropic, due to the application of conventional heat treatment in the lab resistance furnace. In order to gain better performance of the magnets, the annealing of the amorphous precursor has to be carried out in the external magnetic field to induce anisotropy. Therefore, our further studies will be focused on this point.

## Figures and Tables

**Table 1:** The *T_C_* determined for ribbons, 0.5 mm and 1 mm plates in as-cast state and after annealing at various temperatures (*T_a_*—annealing temperature). (Δ*T_C_* = ±3 K).

*T_a_*	*T_C_*_1_ [K]	*T_C_*_2_ [K]
Ribbons		
as-cast	515	—
923 K	627	690
953 K	—	708
1003 K	—	721
1033 K	—	723
	0.5 mm plate	
as-cast	571	674
953 K	—	693
983 K	—	695
1033 K	—	710
1 mm plate		
as-cast	580	705
953 K	—	712
983 K	—	729
1033 K	—	731

**Table 2:** The criteria of fit: *R_exp_*—*R*-expected, *R_wp_*—*R*-weighted pattern and *GOF*—goodness of fit for the Rietveld refinement of XRD patterns of Pr_9_Fe_50_Co_13_Zr_1_Nb_4_B_23_ alloy samples, annealed at various temperatures.

Sample/*T_a_* [K]	*R_exp_*	*R_wp_*	*GOF*
Ribbon			
923	0.643	0.783	1.218
953	0.672	0.922	1.373
1003	0.669	0.933	1.396
1033	0.703	1.141	1.622
0.5 mm plate			
953	0.808	0.959	1.187
983	0.674	0.922	1.373
1003	0.748	1.005	1.345
1033	0.858	1.264	1.473
1 mm plate			
953	0.723	0.934	1.175
983	0.754	0.976	1.181
1003	0.765	0.983	1.265
1033	0.854	1.125	1.378

**Table 3:** Refined lattice parameters for crystalline phases identified for Pr_9_Fe_50_Co_13_Zr_1_Nb_4_B_23_ alloy samples annealed at various temperatures.

Phase Space Group	Pr_2_(Fe,Co)_14_B *P*4_2_/*mnm*		Pr(Fe,Co)_4_B_4_ *Pccn*		α-Fe *Im*-3*m*	Pr_2_O_3_ *Ia*-3
	*a* [nm]	*c* [nm]	*a* [nm]	*c* [nm]	*a* [nm]	*a* [nm]
initial value	0.8811	1.2231	0.7117	3.50699	0.2868	1.1156
***T_a_*** **[K]**	**Ribbon**					
923	0.8763 ± 0.0001	1.2159 ± 0.0002	0.7103 ± 0.0003	3.5056 ± 0.0028	—	—
953	0.8754 ± 0.0001	1.2125 ± 0.0002	0.7104 ± 0.0001	3.4773 ± 0.0013	0.2870 ± 0.0001	—
1003	0.8763 ± 0.0001	1.2141 ± 0.0002	0.7115 ± 0.0001	3.4860 ± 0.0009	0.2868 ± 0.0002	—
1033	0.8764 ± 0.0001	1.2130 ± 0.0002	0.7120 ± 0.0001	3.4856 ± 0.0012	0.2867 ± 0.0001	1.1187 ± 0.0001
	**0.5 mm plate**					
953	0.8757 ± 0.0001	1.2148 ± 0.0002	0.7091 ± 0.0002	3.4705 ± 0.0028	—	—
983	0.8754 ± 0.0001	1.2125 ± 0.0002	0.7104 ± 0.0001	3.4773 ± 0.0013	—	—
1003	0.8769 ± 0.0001	1.2150 ± 0.0002	0.7106 ± 0.0001	3.4855 ± 0.0016	0.2873 ± 0.0003	—
1033	0.8783 ± 0.0001	1.2174 ± 0.0003	0.7126 ± 0.0002	3.4915 ± 0.0014	0.2875 ± 0.0001	1.1165 ± 0.0001
	**1 mm plate**					
953	0.8759 ± 0.0001	1.2143 ± 0.0002	0.7107 ± 0.0001	3.4748 ± 0.0010	—	—
983	0.8757 ± 0.0001	1.2133 ± 0.0002	0.7106 ± 0.0001	3.4768 ± 0.0012	0.2871 ± 0.0001	—
1003	0.8764 ± 0.0001	1.2130 ± 0.0002	0.7104 ± 0.0001	3.4854 ± 0.0018	0.2867 ± 0.0001	—
1033	0.8788 ± 0.0001	1.2151 ± 0.0002	0.7117 ± 0.0001	3.4858 ± 0.0012	0.2868 ± 0.0002	1.1158 ± 0.0001

**Table 4:** Crystallite sizes (*d*) and weight fractions (*Wf*) of constituent phases calculated by the Rietveld method for Pr_9_Fe_50_Co_13_Zr_1_Nb_4_B_23_ alloy samples, annealed at various temperatures.

Phase Space Group	Pr_2_(Fe,Co)_14_B *P*4_2_/*mnm*		Pr(Fe,Co)_4_B_4_ *Pccn*		α-Fe *Im*-3*m*		Pr_2_O_3_ *Ia*-3		Amorphous
	*d* [nm]	*Wf* [%]	*d* [nm]	*Wf* [%]	*d* [nm]	*Wf* [%]	*d* [nm]	*Wf* [%]	*Wf* [%]
***T_a_*** **[K]**	**Ribbon**								
923	20.0 ± 0.3	59.1 ± 0.6	7.9 ± 0.2	15.2 ± 0.3	—	—	—	—	25.7 ± 0.7
953	25.1 ± 0.4	55.9 ± 0.5	12.3 ± 0.5	32.4 ± 0.5	18.4 ± 0.5	11.7 ± 0.3	—	—	—
1003	29.7 ± 0.8	53.2 ± 0.6	15.5 ± 0.4	30.9 ± 0.6	22.4 ± 0.5	14.5 ± 0.3	29.5 ± 2.3	1.4 ± 0.1	—
1033	33.3 ± 1.4	40.9 ± 0.6	17.9 ± 0.7	34.6 ± 0.6	31.5 ± 0.6	20.6 ± 0.4	29.1 ± 1.4	3.9 ± 0.1	—
	**0.5 mm plate**								
953	34.2 ± 0.9	56.3 ± 0.5	14.4 ± 0.8	43.7 ± 0.8	—	—	—	—	—
983	38.3 ± 1.2	59.6 ± 0.6	16.5 ± 0.7	40.4 ± 0.8	—	—	—	—	—
1003	36.3 ± 1.2	51.3 ± 0.6	22.1 ± 0.7	39.4 ± 0.9	7.2 ± 0.3	9.3 ± 0.3	—	—	—
1033	42.3 ± 1.2	46.9 ± 1.4	23.1 ± 0.9	36.2 ± 1.4	12.1 ± 0.7	14.8 ± 1.2	28.7 ± 1.3	2.1 ± 0.1	—
	**1 mm plate**								
953	30.2 ± 0.8	61.3 ± 0.5	14.6 ± 0.6	38.7 ± 0.6	—	—	—	—	—
983	33.3 ± 1.1	51.0 ± 0.7	17.3 ± 0.6	36.9 ± 0.6	7.0 ± 0.4	12.1 ± 0.3	—	—	—
1003	38.3 ± 1.1	44.9 ± 0.7	21.2 ± 0.7	39.2 ± 0.7	8.2 ± 0.5	15.9 ± 0.4	—	—	—
1033	45.2 ± 1.2	42.8 ± 0.7	23.4 ± 0.8	36.8 ± 0.7	12.1 ± 0.5	18.9 ± 1.1	29.2 ± 1.2	1.5 ± 0.1	—

**Table 5:** The magnetic parameters’ values for all samples, annealed at various temperatures (magnetic polarization remanence *J_r_*, saturation polarization *J_s_*, coercivity field *_J_H_c_* and maximum magnetic energy product (*BH*)_max_).

*T_a_* [K]	*J_s_* [T]	*J_r_* [T]	*J_r_*/*J_s_*	*_J_H_c_* [kA/m]	(*BH*)_max_ [kJ/m^3^]
**Ribbon**					
923	0.541	0.377	0.697	762	23
953	0.589	0.398	0.676	656	25
983	0.569	0.344	0.605	562	21
1003	0.628	0.385	0.613	537	17
1033	0.715	0.438	0.612	497	20
**0.5 mm plate**					
953	0.550	0.388	0.705	1130	28
983	0.524	0.367	0.700	1150	25
1003	0.594	0.398	0.670	915	26
1033	0.641	0.412	0.643	795	26
**1 mm plate**					
953	0.535	0.367	0.686	1150	25
983	0.585	0.384	0.656	954	24
1003	0.596	0.380	0.637	865	23
1033	0.647	0.401	0.620	759	23
